# A Prospective Randomized Study: The Usefulness and Efficacy of Negative Pressure Wound Therapy with Lipidocolloid Polyester Mesh Compared to Traditional Negative Pressure Wound Therapy for Treatment of Pressure Ulcers

**DOI:** 10.3390/pharmaceutics12090813

**Published:** 2020-08-27

**Authors:** Wooyeol Baek, Nara Lee, Eun Jin Han, Tai Suk Roh, Won Jai Lee

**Affiliations:** 1 Institute for Human Tissue Restoration, Department of Plastic & Reconstructive Surgery, Severance Hospital, Yonsei University College of Medicine, Seoul 03722, Korea; parande@yuhs.ac (W.B.); nrlee@yuhs.ac (N.L.); rohts@yuhs.ac (T.S.R.); 2 Certified Wound Care Nurse (CWCN), Certified Foot Care Nurse (CFCN), Nurse Department, Severance Hospital, Seoul 03722, Korea; ejhan84@yuhs.ac

**Keywords:** pressure ulcer, negative pressure wound therapy, wound contact layer, Urgotul^®^

## Abstract

To improve healing of pressure ulcer wounds, it is important to optimize the conditions of the area surrounding the wound. Negative pressure wound therapy (NPWT) promotes wound healing, however, the removal of NPWT can cause pain or focal bleeding, delaying wound healing or causing infection. In this study, we reviewed the efficacy of the lipidocolloid non-adherent dressing (Urgotul^®^) as a wound contact layer. A total of 38 patients from the same facility who applied NPWT from April 2016 to October 2019 were included and divided into two groups; NPWT with the lipidocolloid non-adherent dressing (group 1, experimental group, 19 patients) and NPWT only (group 2, control group, 19 patients). The condition of the wound was examined prior to NPWT application, at one week, and again at three weeks after application. No significant differences were found between groups for general characteristics, bacterial culture or photo analysis. However, when comparing groups based on the time of examination, there was a significant reduction of the wound size in group 1 (*p* = 0.001) but not in group 2 (*p* = 0.082). Therefore, the current study finds that using the lipidocolloid non-adherent dressing as a wound contact layer in NPWT stimulates healing by shrinking the size of the pressure ulcer wound.

## 1. Introduction

Pressure ulcers are the result of constant pressure on localized tissue, leading to ischemia of the skin, subcutaneous tissue and muscles surrounding the wound [1]. Localized pressure, friction, shear force, moisture, nutrition, and infection all contribute to the formation of pressure ulcers. Consequently, optimizing the local wound condition to reduce these various issues is an important factor to address during pressure ulcer wound care treatment [2]. Negative pressure wound treatment (NPWT) is a widely used approach for pressure ulcers, helping to promote healing by minimizing intercellular edema and promoting micro-vascularization and granulation tissue formation [3], elimination of excess exudate from wound surface, improvement of blood supply to wound bed, maintenance of adequate wound humidity and reduction of the microorganism burden on the wound surface [4]. However, removal of conventional NPWT can cause pain or focal bleeding, delaying wound healing and possibly causing infection [5]. To address the issues with NPWT removal, various forms of wound contact layers have been developed to reinforce the efficacy of NPWT [6]. One technique used to overcome the issues with removing NPWT has been to apply Vaseline or paraffin gauze on the wound to prevent granulation tissue from being removed when the dressing is changed. However, petroleum jelly disappears over time due to negative pressure, causing the gauze to adhere to the wound and reducing the utility of gauze and petroleum jelly as a method of wound contact protection. Therefore, when using negative pressure therapy for the treatment of pressure ulcers, there is a need to develop alternatives for wound contact layers that will not lead to damage of the newly grown granulation tissue, will not involve pain for the patient, and uses a dressing material that is east to apply.

Urgotul^®^ (Urgo laboratories in Dijon, France) is a non-adherent, polyester mesh dressing made up of hydrocolloid and lipid molecules. When interfacing exudate from the wound, hydrocolloid polymers in Urgotul^®^ absorb it and form lipidocolloid gel. Lipidocolloid wound contact layers help maintain a moist wound environment, helping the wound healing process and reducing pain of dressing removal [6,7]. In addition, when compared with other dressing materials such as gauze or Vaseline, Urgotul^®^ provides the advantage of maintaining a moist environment for an extended period and leaving no residue when replacing the dressing. Several studies [8] have reported that Urgotul^®^ provides an optimal environment for wound healing by affecting cell proliferation and synthesis of the extracellular matrix in the wound area, thereby promoting wound healing and reducing pain [9,10]. We predicted that Urgotul^®^ could serve as a wound contact layer that would effectively promote the wound healing process in pressure ulcers and improve traditional NPWT treatment.

## 2. Materials and Methods

The study protocol followed the tenets of the Declaration of Helsinki and was approved by the Severance hospital institutional review board (IRB 2015-2369-015). The study was designed as a randomized control trial, and all patients gave written, informed consent prior to participation. Forty-nine patients diagnosed with stage 3 or stage 4 pressure ulcers were randomly selected and enrolled in the current study to begin NPWT between April of 2016 and October of 2019. Eleven patients were excluded by the following criteria: (1) pregnant or lactating women and women who have refused to maintain contraception during the clinical period, (2) patients with chronic wasting disease who are not currently receiving stable treatment, (3) patients who have demonstrated hypersensitivity to negative pressure wound therapy or Urgotul^®^, (4) patients who are currently using or who are scheduled to use medication that would have a significant effect on wound healing, (5) patients who the researcher believes would be inappropriate for this study. Thirty-eight patients were confirmed for the final study and received examinations and agreed to voluntary participation. For patients in group 1, Urgotul^®^ was added between the wound and the NPWT as a wound contact layer. For patients in group 2, no wound contact layer was applied. Patients with treatment options other than NPWT were excluded from the study. All measurement processes were carried out according to the study timeline (Table 1).

Braakenburg [11] mentioned that vacuum-assisted closure therapy cannot replace surgical debridement in reducing bioburden. The use of antibiotics and surgical debridement should be complemented by treatment of the infected wound [11]. In the current study, surgical debridement of necrotic tissue was performed based on clinical judgement at the baseline of the study, 1 week and 3 weeks after the treatment.

### 2.1. Size of the Wound

The skin defect area was measured at every dressing change, and compared with the size measured prior to treatment. Measurements were obtained by the VISITRAK^TM^ system for wound area calculation (Smith & Nephew, London, UK). The area was measured by placing a transparent tracing paper on the wound, emulating it along the boundary of the wound, and then measuring it on the VISITRAK^TM^ machine (Smith & Nephew, London, UK).

### 2.2. Granulation Tissue Grade

The granulation tissue grade was evaluated by four professional wound experts at every dressing change. (4: skin intact, 3: granulation tissue consists of 75~100% of total wound area, 2: granulation tissue consists of 25~75% of total wound area, 1: granulation tissue consists of less than 25% of total wound area, 0: granulation tissue consists of 0% of total wound area).

### 2.3. Photo Analysis

Photo analysis was performed by four professional wound experts at three time points: baseline, one week after treatment, and three weeks after treatment. The wound condition was graded on a 1~10 point scale based on general wound condition.

### 2.4. Bacterial Culture

Bacterial culture was taken by the swab culture test at two time points: baseline and three weeks after treatment. We considered reduced number of strains, or reduced number of bacterial colonies as “Improved”, no significant change as “No Interval Change”, and increased number of strains or increased number of bacterial colonies as “Aggravation”.

### 2.5. Statistical Analysis

Results were analyzed with IBM SPSS version 25 IBM Corporation, Armonk, NY, USA). General characteristics of patient data were presented as frequency, percentage, and mean ± SD. The chi-square test, Fisher’s exact test, and independent samples t-test were all performed after verifying homogeneity between the two groups. Bacterial culture results were analyzed using an independent samples t-test. Size, granulation tissue grade and photo analysis were analyzed with a repeated measures ANOVA.

The sample size of the control group and the experimental group was assumed to be a 1:1 ratio. Based on previous reports by Kim [12] the total sample of 38 patients (19 in the control group, 19 in the experimental group) met the criteria for an adequate sample size at a significance level of 5% and at 80% statistical power 14 days after treatment.

## 3. Results

### 3.1. General Characteristics

Thirty-eight total patients were randomly assigned to one of two groups, 19 were assigned to the NPWT with Urgotul^®^ group (group 1, experimental group) and 19 were assigned to the NPWT only group (group 2, control group). The mean age of participants was 60.951240 for group 1, and 60.632138 for group 2. The pressure ulcer wounds of all patients met the criteria for needing NPWT, and all wounds were at stage 4, except for one patient with a stage 3 wound [13]. In group 1, the pressure ulcer wounds were located at the coccyx and sacrum (12 patients), ischium (two patients), trochanter (three patients), back (one patient), and dorsum of the foot (one patient). In group 2, pressure ulcer wounds were located at the coccyx and sacrum (14 patients), ischium (four patients), and trochanter (one patient). The mean period of prevalence of the pressure ulcer wound was 9.261271 months in group 1, 5.68319 months in group 2. The general characteristics and risk factors were similar between two groups (Table 2).

The risk factors for pressure sores in the experimental group were acute phase disease patients (84.2%), elderly people over 65 years old (57.9%), patients with brain damage (42.1%), patients with spinal cord injuries (36.8%), patients with diabetes (36.8%), and patients hospitalized in an intensive care unit (26.3%). The risk factors for pressure sores in the control group were in acute phase disease patients (78.9%), elderly people over 65 years old (52.6%), patients with brain injuries (52.6%), patients with spinal cord injuries (26.3%), patients hospitalized in an intensive care unit (23.3%), and patients with diabetes (21.1%). (Table 2).

Results of a t-test found no statistically significant differences between groups for general characteristics (*p* = 0.248 to 0.1), therefore, the two groups were considered to be homogenous. (Table 2).

### 3.2. Bacterial Cculture

Bacterial culture was taken at the baseline of the study before applying NPWT and again at the last follow-up date. Among the strains identified, *Enterococcus* species was the most common, and MRSA, *Klebsiella, Acinetobacter* and *Pseudomonas* were observed. We considered reduced number of strains, or reduced number of bacterial colonies as ‘Improved’, no significant change as “No Interval Change”, and increased number of strains or increased number of bacterial colonies as “Aggravation”. Results indicated that 57.9% of group 1 and 42.1%, of group 2 met the criteria for “Improved”, 10.5% of group 1 and 26.3% of group 2 met the criteria for “No Interval Change”, and 31.6% of group 1 and 31.6% of group 2 met the criteria for “Aggravation” (*p* = 0.415) (Table 3).

### 3.3. Granulation Tissue Grade

The granulation tissue grade was evaluated by four professional wound experts at every dressing change. (4: skin intact, 3: granulation tissue consists of 75~100% of total wound area, 2: granulation tissue consists of 25~75% of total wound area, 1: granulation tissue consists of less than 25% of total wound area, 0: granulation tissue consists of 0% of total wound area). There was significant improvement in both groups between baseline (T0) and the final follow-up visit (T3). (Table 4.)

### 3.4. Size of the Pressure Ulcer

**Figure 1:**
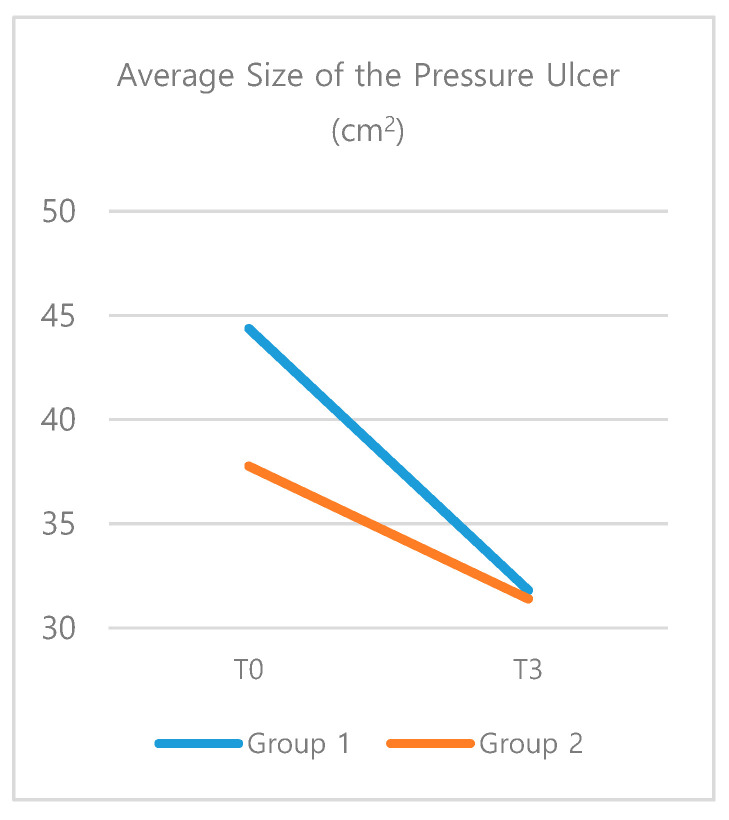
The average size of the pressure ulcer; Results of a two-way ANOVA indicated a significant reduction of the size of the wound in the experimental group (Group 1).

The size of the wound was measured at the baseline (T0), 1 week after NPWT application (T1), and 3 weeks after NPWT application (T3). At T0, there was no significant difference between two groups (*t* = 0.60, *p* = 0.549). There was also no significant difference between two groups based on the results of the repeated-measures ANOVA. However, results of a two-way ANOVA based on group and timing indicated that group 1 had a significant reduction of the size of the wound (T0 M = 44.37, T3 M = 31.81, *p* = 0.001). (Table 4) Group 2 showed no significant difference (*p* = 0.082) (Figure 1).

### 3.5. Photo Analysis

A photo analysis of the wounds was performed by four professional wound experts at baseline (T0), one week after treatment (T1), and three weeks after treatment (T3). Wound condition was graded on a 1~10 point scale based on general wound condition. There was a significant improvement in both groups between T0 and T3, however there was no significant difference between two groups (*p* = 0.694). (Table 4.)

## 4. Discussion

Negative pressure wound therapy is one of the most effective dressing methods for complex wound treatment. NPWT promotes micro-vascularization, aids in granulation tissue formation, and decreases bacterial burden. Patients with chronic wounds with delayed wound healing and those with underlying diseases are typically considered for negative pressure treatment. Despite of the advantages, when changing NPWT dressing, a part of the granulation tissue is detached from the foam contacting area, causing pain or focal bleeding for the patient and delaying wound healing [5,9]. These issues create a need for an alternative suitable wound contact layer between the NPWT and the wound rather than the traditional NPWT that directly contacts the wound. To prevent this, a wound contact layer (WCL) can be used, which is traditionally comprised of Vaseline, silicone sheets or paraffin gauze. The WCL can be used between the wound base and the negative pressure treatment foam to prevent the negative pressure treatment from damaging the wound base. Products suitable for WCL should be able to freely pass wound exudate and pressure through the dressing upon contact with the wound. We hypothesized that Urgotul^®^ could act as an alternative WCL for NPWT for pressure wounds. Bacterial culture, granulation tissue, and wound size were measured to evaluate the wound condition, and photo analysis was performed to compare healing rate [6,14,15].

Urgotul^®^ is a gel-type polyester mesh dressing containing hydrocolloid particles and lipid particles, which is flexible and can be applied to most wounds. When the Urgotul^®^ contacts the exudate of the wound, the hydrocolloid particles in the Urgotul^®^ absorb it to form a lipid-colloidal gel, creating a moist environment suitable for wound healing. Due to the ability of Urgotul^®^ to maintain a long-term wet environment better than traditional gauze or Vaseline without leaving any residue, the aim of this study was to determine if Urgotul^®^ is a more suitable WCL for use with NPWT. In order to assess the effect of Urgotul^®^ as a WCL with NPWT, the culture test, area, and granulation tissue level were evaluated.

No statistical differences were found between the two groups for the bacterial culture test (*p* = 0.415), however it was confirmed that the use of Urgotul^®^ reduced bioburden. NPWT should not be used alone on infected wounds, rather it can be used as an adjunct therapy with antibiotics, debridement, and local antibacterial agents. Shiroky et al. [16] and Benrashid [17] both reported that NPWT could reduce the incidence of surgical wound infection, conversely Costa et al. [18] found no difference in infection rate when comparing negative pressure treatment to use of a normal dressing.

As shown in Braakenburg et al. [11], negative pressure treatment was effective when used in conjunction with antibiotics, aggressive marginal resection, and topical antibacterial agents. The use of WCL with antibacterial agents provides an advantage as the antibacterial effect can be applied to the wound while maintaining the negative pressure treatment effect [14]. Ciliberti et al. [19] reported that antibacterial WCL was effective in combination with negative pressure treatment in a severe bacterial colonization stage pressure sore. Although antibacterial effect was not reported with Urgotul^®^, reduction of bioburden in the experimental group was confirmed. In the group that applied NPWT and Urgotul^®^ together, 11 cases out of 19 cases had a reduction of the number of strains and a decrease in the amount of bacteria, while only two cases did not change. Out of 19 cases in the group using NPWT alone, eight cases showed improvement, and five cases had no reported change. The number of samples in the current study is small, which may have contributed to the lack of statistical significance, therefore further study is needed with larger sample sizes.

Krasner [7] found that using WCL in combination with NPWT was helpful in reducing the size of the wound in addition to improvements in granulation tissue formation which can, in turn, reduce the pain patients experience with wound dressing changes. As new granulation tissue penetrates between the pores of the negative pressure treatment foam, when the dressing is removed the granulation tissue is damaged and patients complain of pain. For this reason, WCL is recommended when granulation tissue growth is expected to be very rapid or when wound contraction is needed quickly [7]. Most pressure wound patients have no sensation, so levels of pain experienced with dressing changes can be challenging to investigate. Even when pain is not a consideration, if damage to the granulation tissue caused by removing NPWT foam can be prevented, it would promote granulation tissue formation and accelerate healing of the wound.

In the current study, there was a significant area change before and after 3 weeks of treatment in the group where Urgotul^®^ and NPWT were applied together (*p* = 0.001), which was not found in the group where only NPWT was applied (*p* = 0.082). Wounds become smaller in size due to intra-wound migration and wound contraction of epithelial cells. NPWT foam tends to block the path of the epithelial cells from moving into the wound as it penetrates the granulation tissue. When used with Urgotul^®^, NPWT foam did not penetrate the granulation tissue or block the epithelial cell interface. Urgotul^®^ seems to have promoted migration into the wounds of the epithelium by protecting the wound from loss of granulation tissue typically blocked by NPWT foam. Furthermore, it was confirmed that Urgotul^®^ is a WCL that does not reduce the effectiveness of NPWT. In granulation tissue grade and photo analysis, both the control group and the experimental group showed statistically significant improvement after 3 weeks of treatment. However, these findings are limited as the wound size calculation in this study did not include the wound margin condition or depth of the wound. The area of the skin defect was the only consideration in size measurement, therefore it was not possible to evaluate the wound condition accurately.

## 5. Conclusions

In conclusion, although NPWT is a very effective method for wound healing, it is possible to reduce the size of wounds faster when NPWT is used in conjunction with non-adherent lipidocolloid polyester mesh as a WCL, without reducing the effectiveness of negative pressure therapy.

## Figures and Tables

**Table 1:** Study timeline. Measurements were taken at three different time points. Physical examination and evaluation of the wound were performed according to the corresponding protocol at each time point.

**Screening Period (1~14 days before Treatment)**
(1) Physical examination and confirm selection/exclusion criteria based on records (2) Bacterial culture taken from the pressure ulcer wound (3) Gross photo taken (4) Evaluate wound size, depth, and condition (5) Present study concept (6) Perceive consent from the patients
**Treatment period (the day of Starting Treatment)**
(1) General treatment process follows conventional NPWT in all patients (2) Debridement of wound and wound around area necrotic tissue (3) Gross photo taken (4) Evaluate wound size, depth, and condition (5) In Group 1, Urgotul^®^ was applied to the size of the wound (6) NPWT dressing applied to the size of the wound
**Follow Period (~3 Weeks after the Treatment)**
(7) Dressing change twice a week (8) Gross photo taken at 1 week after the treatment (9) Evaluate wound size, depth, and condition every week (10) Bacterial culture taken on every dressing change

**Table 2:** General characteristics; the characteristics of patients were simply classified. There were no significant differences between the two groups. Of the initial 49 patients, 11 were excluded, and the remaining 38 patients participated in the study.

Characteristics	Categories	Exp. (n = 19)	Cont. (*n* = 19)	*t* or x^2^	*p*
		M ± SD/n (%)	M ± SD/n (%)		
**Gender**	Male	10 (52.6)	12 (63.2)	0.43	0.511
	Female	9 (47.4)	7 (36.8)		
**Age (years)**		60.95 ± 12.40	60.63 ± 21.38	0.56	0.956
**Period of prevalence (months)**		9.26 ± 12.17	5.68 ± 3.19	1.19	0.248
**Location of pressure sore**	Coccyx	12 (63.2)	14 (73.7)	3.82	0.431
	Ischium	2 (10.5)	4 (21.1)		
	Trochanter	3 (15.8)	1 (5.3)		
	others	2 (10.6)	0 (0)		
**Stage of pressure sore**	Stage 3	1 (5.3)	0 (0)	1.03	0.311
	Stage 4	18 (94.7)	19 (100)		
**Risk factors**	Age over 65	11 (57.9)	10 (52.6)	0.11	0.744
	BMI (kg/m^2^)	19.55 (4.2)	20.38 (4.6)	0.58	0.570
	Spinal cord injury	7 (36.8)	5 (26.3)	0.49	0.485
	Brain damage	8 (42.1)	10 (52.6)	0.42	0.516
	Acute phase disease	16 (84.2)	15 (78.9)	0.18	0.656
	DM	7 (36.8)	4 (21.1)	1.15	0.283
	ICU admission	5 (26.3)	5 (26.3)	0.00	1.000

*n* = 38; Cont. = Control group; Exp. = Experimental group; M = Mean; SD = Standard deviation.

**Table 3:** Bacterial culture count result; Various strains were identified for each individual and categorized to reflect changes in the number of bacteria before and after NPWT.

Bacterial Count	Group 1 (n = 19)	Group 2 (n = 19)	Group1 (*p*)	Group2 (*p*)
	N (%)	N (%)		
Improved	11 (57.9)	8 (42.1)	1.76	0.415
No interval change	2 (10.5)	5 (26.3)		
Aggravation	6 (31.6)	6 (31.6)		

**Table 4:** Wound Evaluation; The results of changes in pressure wound size, granulation tissue grade, and photo analysis evaluated by experts before and after treatment were compared. Each evaluation showed a significant improvement in both groups (N = 38).

Variable	Group	T0	T3	Source	F	*p*
		M (SD)	M (SD)			
Size	Exp. (N = 19)	44.37 (± 7.79)	31.81 (± 5.43)	G	0.15	0.704
		Md = 12.56, *p* = 0.001		T	14.13	0.001
	Cont. (N = 19)	37.77 (± 7.79)	31.40 (± 5.43)	G × T	1.51	0.227
		Md = 6.37, *p* = 0.082				
Granulation tissue grade	Exp. (N = 19)	1.29 (± 0.12)	3.58 (± 0.15)	G	0.51	0.478
		Md = –2.29, *p* ≤ 0.001		T	567.21	< 001
	Cont. (N = 19)	1.15 (± 0.116)	3.49 (± 0.153)	G × T	0.07	0.778
		Md = –2.34, *p* ≤ 0.001				
Photo analysis	Exp. (N = 19)	3.26 (± 0.15)	4.63 (± 0.22)	G	0.05	0.820
		Md = –1.37, *p* ≤ 0.001		T	114.95	< 001
	Cont. (N = 19)	3.16 (± 0.15)	4.63 (± 0.22)	G × T	0.16	0.694
		Md = –1.47, *p* ≤ 0.001				

Cont. = Control group; Exp. = Experimental group; G = Group; M = Mean; SD = Standard deviation; T = Time; T0 = Pretest; T3 = 3 week Follow up test after T0 test. *p* < 0.05.
